# Isometric Arm Strength and Subjective Rating of Upper Limb Fatigue in Two-Handed Carrying Tasks

**DOI:** 10.1371/journal.pone.0119550

**Published:** 2015-03-20

**Authors:** Kai Way Li, Wen-Sheng Chiu

**Affiliations:** 1 Department of Industrial Management, Chung Hua University, Hsin-Chu, Taiwan; 2 Ph.D Program of Technology Management, Chung Hua University, Hsin-Chu, Taiwan; University of Rome Foro Italico, ITALY

## Abstract

Sustained carrying could result in muscular fatigue of the upper limb. Ten male and ten female subjects were recruited for measurements of isometric arm strength before and during carrying a load for a period of 4 minutes. Two levels of load of carrying were tested for each of the male and female subjects. Exponential function based predictive equations for the isometric arm strength were established. The mean absolute deviations of these models in predicting the isometric arm strength were in the range of 3.24 to 17.34 N. Regression analyses between the subjective ratings of upper limb fatigue and force change index (FCI) for the carrying were also performed. The results indicated that the subjective rating of muscular fatigue may be estimated by multiplying the FCI with a constant. The FCI may, therefore, be adopted as an index to assess muscular fatigue for two-handed carrying tasks.

## Introduction

Manual materials handling (MMH) tasks are major contributors of musculoskeletal injuries on workplaces [1]. They are the sources of financial burden for industries in terms of lost work days and worker compensation costs [2, 3]. Determining physical capability for workers has been a fundamental issue in job design for manual tasks [4–6].

Muscular strength has been one of the major predictors assessing physical capability. It is the maximum force output for a certain muscle group or for body segments under specific posture and force exertion conditions. It decreases when the force exertion lasts for a period of time due to muscular fatigue. Muscular fatigue is the phenomenon of failing to maintain the required force [7] or to continue working at a given exercise intensity [8]. A decrease of muscular strength or force output is observed when muscular fatigue occurs [9–11]. Muscular fatigue may, therefore, be regarded as a synonym of “decrease of muscular strength” even though other sources such as lack of motivation and musculoskeletal injury [11, 12] could also contribute to muscular strength reduction. Muscular fatigue may relate to the risk of overexertion and could contribute to musculoskeletal injuries directly or indirectly [13, 14].

Assessment of muscular fatigue may be performed via measuring reduction of maximum voluntary contraction (MVC) or force output after performing a forceful exertion. When measuring the reduction of the MVC, a force exertion lasting for a certain period is required. The reduction of the MVC is measured using a force gauge. Mathematic functions such as exponential ones may, then, be fitted as models to describe the developing of muscular fatigue. Scientists, utilizing such an approach, have established muscular fatigue models for various task conditions [15–18].

Roman-Liu et al. [17] proposed a force change index (FCI) to differentiate muscle fatigue on upper limb for subjects performing intermittent hand grip tasks. The FCI was calculated using the following equation:

$$\text{FCI }=\frac{f(t_{2})-f\left(t_{1}\right)}{f(t_{1})}$$ (1)

where *f*(*t*) is the estimated force value based on a regression equation of change of relative exerted force over time, *t*_*1*_ and *t*_*2*_ are the time at the beginning and end of loading, respectively. The FCI has been a recommended as a valid index to assess muscle fatigue for handgrip task and to differentiate the difference in fatigue resulting from differences in the external load.

An exponential model has been adopted to describe the developing of muscular fatigue in single arm pushing tasks [12, 19–21]. This model adopts a fatigue parameter, or fatigue rate (*k*), to indicate the physical attribute of either an individual or a certain population. This parameter is determined by factors such as muscle group, muscle fiber composition, age, gender, and so on. Zhang et al. [21] assessed muscular fatigue for workers in an electronic factory. They found that gender was a significant (*p*<0.0001) factor affecting fatigue rate and males had significantly higher fatigue rate than those of females. Ma et al. [12, 19, 20], on the other hand, used this parameter to discuss fatigue of upper limb for drilling tasks in air craft assembling operations. The exponential model for muscular strength may be described in the following equation:

$$F\left(t\right)=MVCe^{-k\frac{L_{}}{MVC}t}$$ (2)

or alternatively,

$$\text{ln}\left(\frac{F\left(t\right)}{MVC}\right)=-k\frac{L_{}}{MVC}t$$ (3)

where *F*(*t*) is the muscular strength at time *t* after performing a physical task, *MVC* is the maximum voluntary contraction of the muscle, *L* is the muscle force required to balance the external load, and *k* is the fatigue rate. Equation (3) may be represented as Equation (4) if *y* is equal to $\text{ln}\left(\frac{F\left(t\right)}{MVC}\right)$ and b is equal to-*k L/MVC*.

y= b×t (4)

The regression coefficient b may be obtained if both *F*(*t*) and *MVC* are known. The fatigue parameter *k* may, then, be determined as-b×*MVC/L* and Equation (2) may, then, be directly applicable.

Measuring & modeling the trends of muscular strength are beneficial to enhance our understanding on developing of muscular fatigue and job design concerning MMH tasks [22]. When measuring the isometric arm strength, the subject stands erect with upper arms straight down and lower arms in horizontal. This posture is the same as when people are carrying a container using both hands in the front. The isometric arm strength may, therefore, be regarded as a predictor of carrying strength. The purposes of this study were to discuss levels of muscular fatigue when performing two-handed carrying tasks via examining the isometric arm strength, the FCI, and subjective rating of upper limb fatigue.

## Methods

An experiment was conducted to measure the two-handed carrying strength after carrying a weight for a period of time. The isometric arm strength, measured with the same posture as when people carry an object using two hands in front of the abdomen, was measured. The study was approved by an Ethics Committee of the Chung Hua University. All subjects signed informed consent.

### Instrument

A force gauge (Lutron Inc., FG-5100) was adopted to measure the isometric arm strength of the subject. This gauge and the attached S-shape loadcell were connected to a chain. This connection allows measurements of pulling force on the chain. The force gauge is capable of measuring a force up to 980 Newtons (N). A digital display on the gauge indicates the maximum force in each trial.

### Human Subjects

Ten males and ten females were recruited as human subjects. All the subjects did not have musculoskeletal disorders in the past. The age, stature, body mass, and body mass index (BMI) for the female subjects were 20.30 (±1.06) yrs, 160.65 (±4.70) cm, 54.90 (±6.03) kg, and 21.27 (±2.21) kg/m^2^, respectively. The age, stature, body weight, and body mass index (BMI) for the male subjects were 23.80 (±2.20) yrs, 171.50 (±4.77) cm, 71.10 (±10.50) kg, and 24.12 (±2.86) kg/m^2^, respectively. All the subjects were requested to refrain from sports or strenuous physical activities the same day before joining the experiment.

### Weight handled, and sustained time

A commercially used plastic container was adopted (50 cm × 36 cm × 27 cm). The container has handles (2 cm × 3.5 cm) on the sides. The handles were wrapped with sponge of approximately 1 cm thick so as to reduce the discomfort of the hands in the sustained carrying task. The subject carried a container in front of his/her abdomen with his/her upper arm straight down and his/her lower arm in horizontal. Metal blocks were evenly paced in the container to comprise the weight to be handled. The total mass of the metal blocks and the container was either 4 kg or 8 kg for female subjects and 5 kg or 10 kg for male subjects, respectively. These loads were common in the manual materials handling tasks in industry. The container was carried by the subject for four minutes.

### Dependent variables

**Fig 1:**
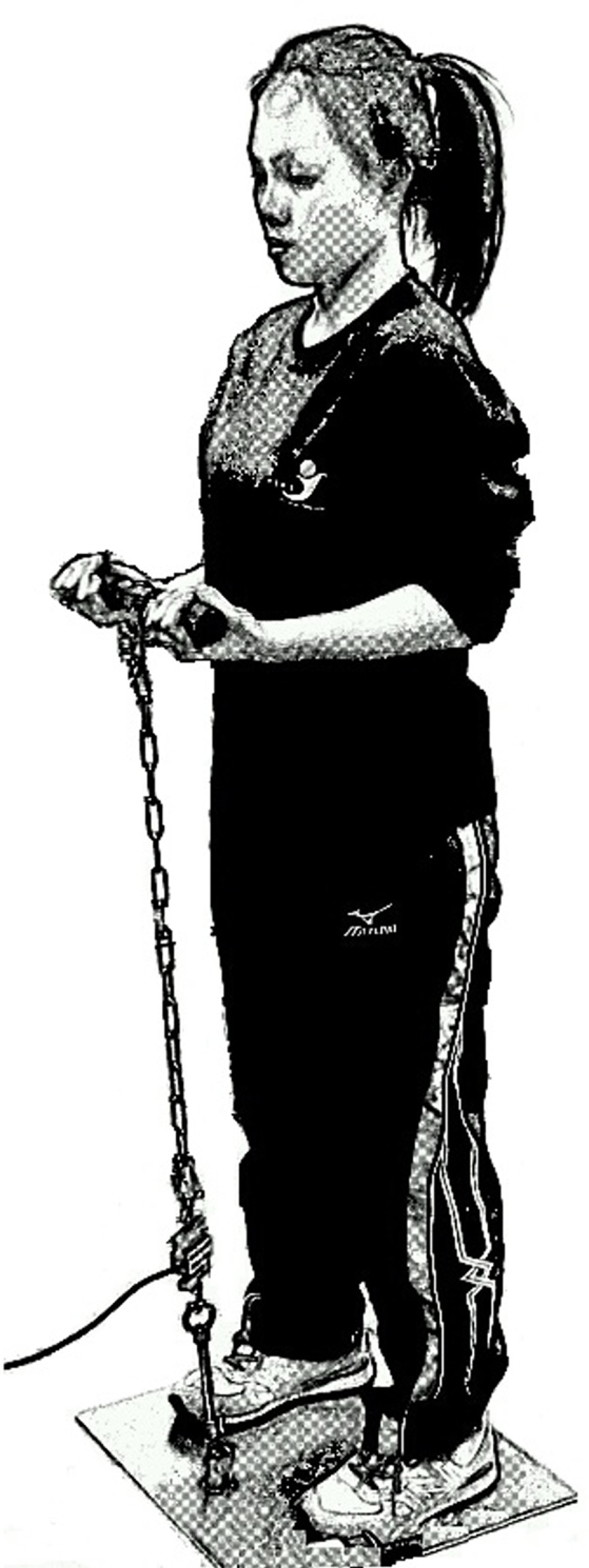
Measuring the isometric arm strength.

The dependent variables of the experiment included isometric arm strength, FCI, and subjective rating of upper limb fatigue. When measuring the isometric arm strength, the subject stood straight on a steel plate. A chain, connecting to a loadcell, was anchored to the steel plate. The handle on the top of the chain was at the elbow height of the subject. The subject pulled the handle upward when his/her upper arm was straight down and by the sides (see Fig. 1). The subject pulled as hard as he/she could and continued for 4 to 6 seconds [23]. The maximum force during this period was recorded as *F*(*t*). This force is the maximum force output the subject could sustain under a normal two-handed carrying posture. Based on Equation (1) & (2), the modified FCI using the following equation was defined:

$$\begin{array}{l} \text{FCI }=\left|\frac{f(t_{2})-f\left(t_{1}=0\right)}{f(t_{1}=0)}\right|\\ =\left|\frac{MVCe^{-k\frac{L_{}}{MVC}t_{2}}-MVCe^{0})}{MVCe^{0}{}_{}}\right|\\ =\left|e^{-k\frac{L_{}}{MVC}t_{2}}-1\right| \end{array}$$ (5)

where *t*_*2*_ is 0.5, 1, 1.5, .., or 4.

The subjective ratings of muscular fatigue of the upper limb were measured using the Borg CR-10 scale [24].

### Experiment Procedure

**Fig 2:**
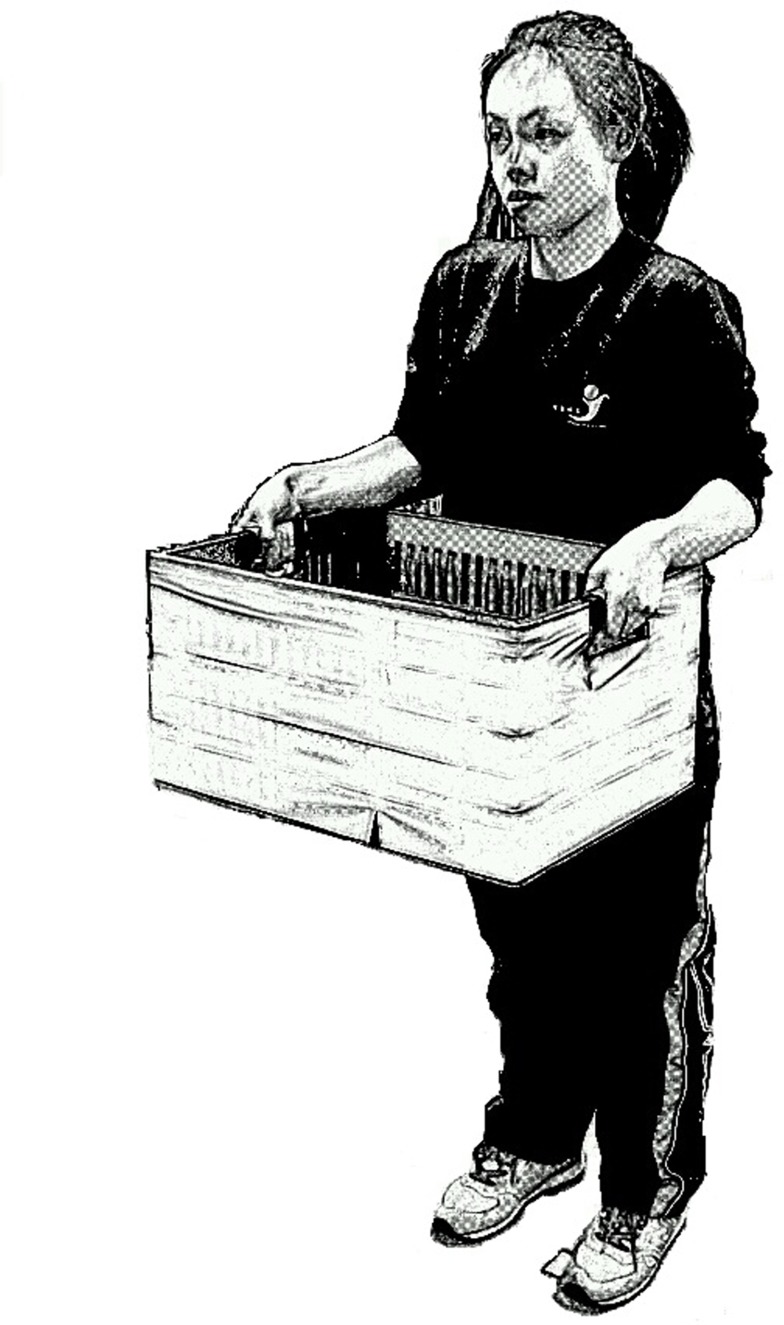
Subject carrying a container.

The isometric arm strength measured at the beginning of a session was the MVC in Equation (2) for the carrying posture which might be regarded as the carrying or holding strength of the subject when there is no fatigue effect. After this measurement, each subject was required to lift and carry a container with one of the mass conditions assigned using both hands for 4 minutes (see Fig. 2). For every half minutes (*t* = 0.5, 1, .., 4 min), the subject put down the container to have the isometric arm strength (*F*(*t*)), and subjective ratings of upper limb fatigue measured. After these measurements, the subject continued the carrying task. This process continued till the end of the four minute test period. A total of nine isometric arm strengths, including the MVC, and eight subjective ratings of upper limb fatigue were collected for each trial. Upon completion of the trial for one handling condition, the subject was dismissed and was requested to return at least one day later for the next handling condition.

### Experiment design and data analysis

Each subject attended two experimental sessions according to the mass to be handled. The level of mass handled was determined randomly. There were a total of 360 (2 levels of mass × 9 strength measures × 20 subjects) isometric arm strength measurements and 320 (2×8×20) subjective rating on muscular fatigue measurements. Descriptive statistics, analysis of variance (ANOVA), and regression analyses were performed. Statistics were conducted using the SAS 9.0 software.

## Results

### Decrease of Isometric strength for carrying

The MVC of the isometric arm strength for male and female subjects were 330.78 (±48.31) N and 201.99 (±26.00) N, respectively. The difference between the two genders was statistically significant (*p*<0.0001). The isometric arm strengths for male subjects were approximately 1.6 times of those of the females. The MVC data were from the readings of the force gauge under the posture and conditions shown in Fig. 1. The corresponding torques for the two genders were 113.76 (±19.58) Nm and 64.08 (±8.66) Nm, respectively. The torque was calculated by multiplying the reading of the force gauge with the length of the moment arm, or the distance from the elbow joint to the handle. The distances from the elbow joint to the point of application of force for male and female subjects were 0.34 (±0.02) m and 0.32 (±0.02) m, respectively. The torque (Nm) data were approximately one third of those of the force (N).

**Fig 3:**
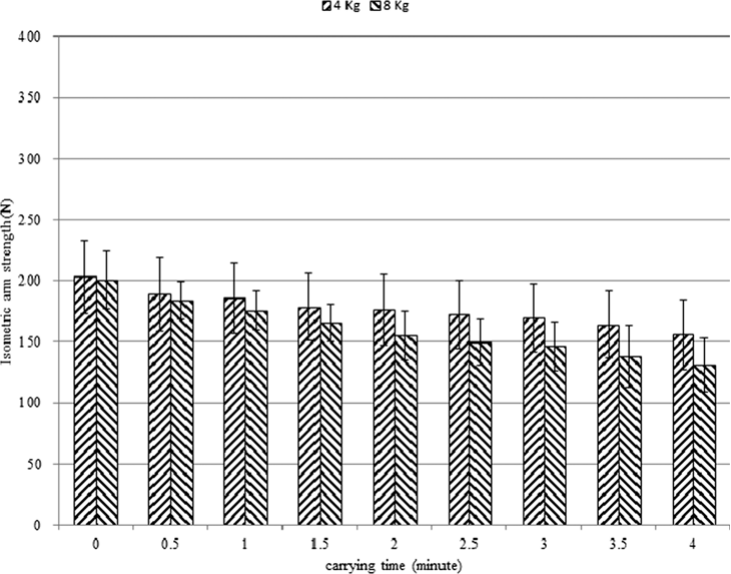
Isometric arm strength for female subjects.

**Fig 4:**
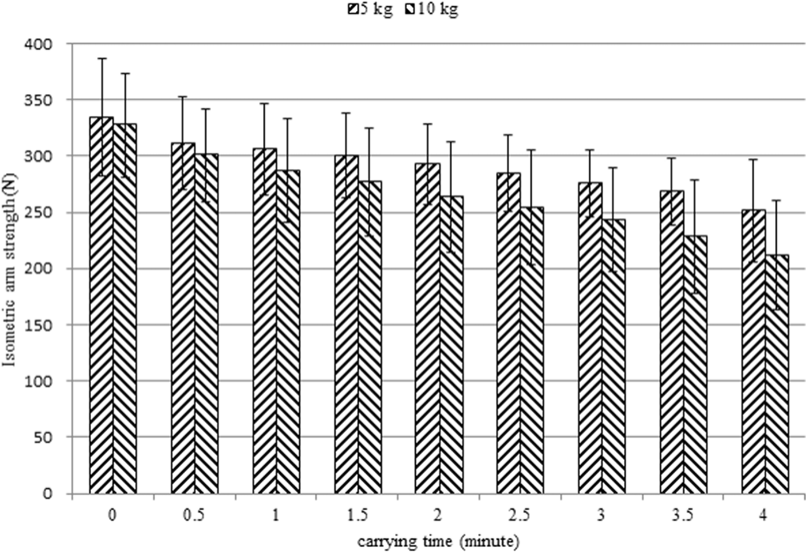
Isometric arm strength for male subjects.

Figs. 3 & 4 show isometric arm strength for female and male subjects, respectively. Sustained carrying resulted in reduction of strength. The remaining isometric arm strengths (%) at the end of the trial were determined by dividing the isometric arm strength at *t* = 4 by the MVC. For female subjects, the remaining isometric arm strengths (%) at the end of the trial of the 4 kg and 8 kg conditions were 77% and 65%, respectively. For male subjects, the remaining isometric arm strengths (%) under the 5 kg and 10 kg conditions were 75% and 65%, respectively.

### Determining fatigue parameter

As the decreases of the isometric arm strength after completing the carrying tasks were observed, regression analyses were conducted fitting Equation (4) (or *y* = *b*×*t*) based on the *F(t)* and *MVC* for each load carrying condition for each subject. The coefficient of determinations (*R* ^*2*^) [25] of the regression models for females were in the range from 0.89 to 0.99 and for males of 0.84 to 0.99, respectively. The fatigue parameter *k* for each carrying condition for each subject was also calculated. Table 1 shows the fatigue parameter *k* values under experimental conditions. ANOVA tests were conducted testing the effects of weight handled on *k* for both the male and female subjects. Both results were not significant.

**Table 1:** Fatigue parameter *k*.

	Female		Male	
	4 kg	8 kg	5 kg	10 kg
Mean	0.34	0.29	0.47	0.36
standard deviation	0.14	0.15	0.28	0.78

### Predicting carrying strength for individual

As the *k* values were determined, Equation (2) may be used to predict the carrying strength for each individual or a group. For example, the *MVC* and *k* were averaged 327.21 N and 0.357 min^-1^, respectively, for the ten male subjects carrying 10 kg mass (29.9% MVC equivalent). Substitute these values in Equation (2), we have:

$$\begin{array}{l} f\left(t\right)=MVCe^{-k\frac{L_{}}{MVC}t}\\ =327.21e^{\left(-0.357\times 10\times 9.8/327.21\right)t}\\ =327.21e^{-0.107t} \end{array}$$ (5)

**Fig 5:**
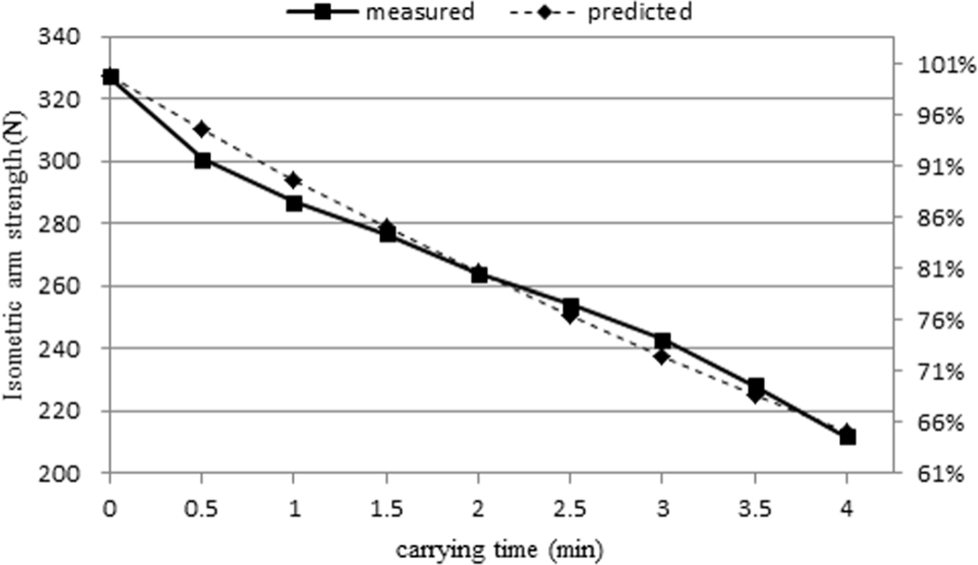
Measured and predicted isometric strength of male subjects carrying a 10 kg mass.

Fig. 5 shows the measured and predicted isometric strength of these male subjects. Comparing the isometric arm strength at *t* = 4 and at *t* = 0, the isometric arm strength dropped approximately 36% for both the measured and predicted strengths.

**Fig 6:**
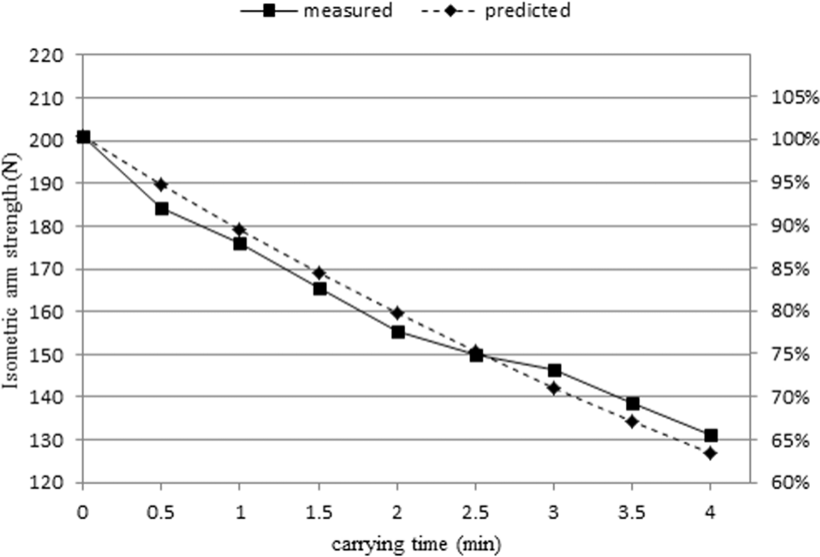
Measured and predicted isometric arm strength for female subjects carrying an 8 kg mass.

Fig. 6 shows the decrease of isometric arm strength for the ten female subjects carrying a mass of 8 kg. The averaged MVC and the *k* for these subjects were 200.87 N and 0.294 min^-1^, respectively. The predicted isometric arm strengths were calculated using Equation (6). The isometric arm strengths dropped approximately 35% and 37% for measured and predicted strengths, respectively, in the 4-minute carrying task.

$$\begin{array}{l} F\left(t\right)=MVCe^{-k\frac{L}{MVC}t}\\ =200.87e^{\left(-0.294\times 8\times 9.8/200.87\right)t}\\ =200.87e^{-0.115t} \end{array}$$ (6)

For each of the subject, predictive equations could be obtained in a similar way just mentioned. A mean absolute deviation (MAD) was defined to compare the difference between the measured and predicted carrying strength after carrying a weight for a time period *t* using Equation. (7). The MAD could also be presented in percentage if the right-hand side of Equation. (7) is divided by measured value and multiplied by 100% and was termed MADP(%). The predicted values were calculated using the individual predictive equation for each weight condition for each subject. The MAD and MADP(%) for the time period *t* were shown in Table 2. Each of the value in the table was the average of 10 subjects.

$$\text{MAD}=\frac{1}{n}\overset{n}{\underset{i=1}{\sum^{\text{​}}}}\left|\text{measured}\;\text{value}-\text{predicted}\;\text{value}\right|$$ (7)

**Table 2:** MAD (N) and MADP(%) for male and female subjects.

	time	0.5	1	1.5	2	2.5	3	3.5	4
Male	5 kg	13.54	10.11	9.35	10.10	7.66	5.71	5.75	13.26
		4.28	3.35	2.96	3.31	2.58	2.08	2.17	4.98
	10 kg	14.93	17.34	13.95	10.29	7.24	5.86	8.45	7.45
		5.08	6.48	5.31	3.94	2.86	2.54	3.77	3.84
Female	4 kg	9.57	5.69	7`.78	4.24	3.26	3.56	3.99	4.62
		5.22	3.22	4.54	2.39	2.00	2.02	2.39	3.01
	8 kg	7.92	6.11	7.02	5.72	4.23	3.24	4.63	5.85
		4.14	3.48	4.32	3.91	2.90	2.32	3.64	4.56

### FCI & subjective ratings on muscular fatigue

The FCI and subjective ratings of upper limb fatigue values for the time period were summarized in Tables 3 & 4, respectively. The Pearson’s coefficient of correlation between FCI and subject rating was 0.71 (*p*<0.0001). As the subjective rating of muscular fatigue is an indirect measure of muscular fatigue, linear regressions of this variable on FCI without intercept were conducted. The results were shown in Table 5.

**Table 3:** Mean and (±) standard deviation of FCI.

	time	0.5	1	1.5	2	2.5	3	3.5	4
Female	4 kg	0.033	0.064	0.095	0.124	0.152	0.179	0.205	0.231
		±0.012	±0.023	±0.034	±0.043	±0.053	±0.062	±0.069	±0.077
	8 kg	0.054	0.105	0.152	0.197	0.238	0.277	0.314	0.348
		±0.025	±0.047	±0.067	±0.084	±0.099	±0.112	±0.124	±0.134
Male	5 kg	0.033	0.065	0.095	0.124	0.152	0.179	0.205	0.230
		±0.014	±0.028	±0.040	±0.052	±0.062	±0.072	±0.081	±0.089
	10 kg	0.053	0.103	0.150	0.195	0.237	0.277	0.314	0.349
		±0.014	±0.026	±0.037	±0.046	±0.055	±0.062	±0.069	±0.075

**Table 4:** Mean and (±) standard deviation of subjective rating of muscular fatigue.

	time	0.5	1	1.5	2	2.5	3	3.5	4
Female	4 kg	2.30	2.90	3.40	3.70	3.90	4.30	4.50	4.70
		±0.82	±0.74	±0.52	±0.82	±1.10	±1.34	±1.18	±1.57
	8 kg	4.20	4.60	5.10	5.80	6.50	6.90	7.50	7.80
		±1.69	±1.58	±1.45	±1.55	±1.51	±1.37	±1.18	±1.23
Male	5 kg	1.60	2.00	2.40	3.00	3.60	4.10	4.80	5.50
		±0.92	±0.99	±1.14	±1.37	±1.43	±1.70	±1.63	±1.81
	10 kg	2.20	2.90	4.20	4.90	5.40	6.30	7.00	7.80
		±0.92	±0.99	±1.14	±1.37	±1.43	±1.70	±1.63	±1.81

**Table 5:** Regression coefficients & *R* ^*2*^ for subjective rating of muscular fatigue over FCI considering gender & load carrying conditions.

		regression coefficient*	*R* ^*2*^
Female	4 kg	21.23	0.73
	8 kg	23.37	0.84
	combined	22.75	0.81
male	5 kg	22.34	0.91
	10 kg	22.62	0.92
	combined	22.53	0.92
all subjects	all loads	22.64	0.86

*all the regression coefficients were statistically significant at *p*<0.0001.

## Discussion

The isometric arm strengths before and during the carrying tasks were measured. The posture in measuring the isometric arm strength was the same as that in carrying a container in the front except that the wrist positions were somewhat different. This posture is mainly associated with elbow flexion and elbow flexors are the predominant muscles in producing the force output. The biceps brachii, brachialis and brachoradialis muscles all contribute to elbow flexion and their relative contributions are different under the two wrist positions in Figs. 1 and 2, respectively. The isometric arm strength may be regarded as a capacity measure of these elbow flexors under the specific postures in Fig. 1 and the decrease of this strength may be regarded as the developing of muscular fatigue in these muscles. It should also be noted that only one MVC value was collected for each trial for each subject. As multiple measures were recommended [26] to increase the reliability of the data when measuring the MVC, our MVC values could either be true MVCs or approximations of the MVCs.

In industries, most force requirements are not adjusted to each individual but controlled at fixed levels. Instead of using a fixed ratio of %MVC, the weights of the container were adopted to generate external force in the static carrying posture. These weights could result in different %MVC levels for different subjects. The %MVC levels for female subjects were in the ranges of 15%~25% and 32%~43% for the 4 kg & 8 kg handling conditions, respectively. For male subjects, they were in the ranges of 11%~18% and 24%~36% for the 5 kg & 10 kg conditions, respectively. These ranges cover moderate levels of materials handling tasks in industry. The fatigue parameter *k*-based exponential models might well describe the decrease of the isometric arm strength. A four-minute carrying of the tested loads resulted in averaged FCI values from 0.23 to 0.49 and averaged Borg CR-10 ratings of muscular fatigue from 4.7 (moderate) to 7.8 (very strong).

Roman-Liu et al. [17] indicated that the FCI can be applied to assess muscular fatigue as it differentiate the difference of upper limb fatigue resulting from the differences in external load. The two levels of mass carried by the male and female subjects were different. However, Table 3 shows that the FCI levels for the two genders were almost equivalent both at the low (4 kg & 5 kg for female and male subjects, respectively) and high levels (8 kg & 10 kg for the two genders, respectively) of mass carried. This implied that the male subjects were experiencing the equivalent burden as that of the female subjects when they were carrying a weight 1.5 times higher than that of the females. The regression results in Table 5 indicate that the linear relationship between the subjective rating of upper limb fatigue and FCI was also equivalent between male and female subjects as the regression coefficient of the male model (22.53) was close to that of female model (22.75). Neither was the weight handled a significant factor. The models indicate that the subjective rating of upper limb fatigue may be obtained by multiplying the FCI with a constant (the regression coefficient). The literature [17] has proposed that the FCI could be applied to assess muscular fatigue for intermittent hand-grip tasks. This study showed that it may also be used to quantify muscular fatigue for two-handed carrying tasks.

There are limitations of this study. First, the isometric arm strengths at different time periods were measured chronologically instead of randomly. The strength measurements at early stages could result in additional fatiguing effects on the later measurements. Such effects could not be split with the effects of the carrying. In addition, the embedded arm strength measurements could also provide short breaks (even in seconds) during sustained carrying which could alter the trend of muscular strength decrease. Third, only two levels of load carried for each of male and female subjects were tested. This resulted in limited range of force exertion. Future research may be required to extend the range of load carrying so as to increase our understanding of muscular fatigue for two-handed load carrying tasks.

## Conclusion

An experiment concerning two-handed carrying tasks for male and female subjects was conducted. Isometric arm strengths of the subjects were collected before and during the four-minute weight carrying tasks. Via incorporating the fatigue parameter *k*, predictive equations for isometric arm strength along the time period of weight carrying were established. Such predictive equations may be adopted to predict the trends of isometric arm strength for an individual and for male and female populations when performing two-handed carrying tasks. Linear regression equations were established between the Borg-CR 10 based subjective rating for muscular fatigue and FCI for the weight carrying tasks with acceptable *R* ^*2*^. The results of the regression analyses indicated that the FCI may be adopted as an index to assess muscular fatigue for two-handed carrying tasks. This is consistent with the findings of Roman-Liu [17].
